# Report of a Case of Puerperal Convulsions

**Published:** 1864-06

**Authors:** Robert Taylor

**Affiliations:** Pendleton, Ohio


ART. II.—*Report of a case of Puerperal Convulsions*:—By Robert Taylor, M. D.

Pendleton, Ohio.

April 24.—Dr. Day and myself were called to see Mrs. Lieut. A. A——, aged 22, of a nervo-sanguinolent temperament; having been found early in the morning, in her chamber, greatly agitated and unconscious. Shortly after, she was attacked with a convulsion, succeeded by several more before our arrival. When first seen, she was in a great state of nervous excitement, delirious, not recognizing any one, pulse slow, full and labored. I immediately bled her from the arm. After the abstraction of twenty-five ounces, the pulse became less labored and more natural. Cold applications were now applied to the head, and sinapis foot-baths and other warm applications used to the lower extremities.

The above treatment did not, however, in the least arrest the frequency of the convulsions. We now resorted to the inhalation of chloroform, then chloroform and sul. ether in combination, one part of the former to two parts of the latter. There was no return of convulsions during the time the patient was under an anæsthetic, which was continued for sixteen hours, with a single exception. This time we allowed the anæsthetic influence to pass off, hoping that the convulsions might not return. But in this we were disappointed, as a convulsion came, succeeded by a second, before we had time to arrest them, by the only treatment that proved efficient in our hands.

Labor was progressing naturally, and under ordinary circumstances favorably. Instrumental interference was talked of, for the purpose only of terminating labor rapidly. But after due consideration, we concluded to allow nature to complete the work; as there was a cephalic presentation and no pelvic deformity.

April 25th.—The child was born at 3 A. M. Chloroform, now was discontinued, as the exciting cause was removed, to-wit: the pressure of the child on the pelvic nerves.

After the anæsthesia had passed off, the patient presented the same restlessness, nervous excitement and unconsciousness which were present when first seen. These phenomena were soon followed by two convulsions in quick succession, which were again arrested by the partial effects of chloroform and the administration of a full dose of morphia sulphas, followed in a half an hour by the fluid extract of valerian ʒi. Here the convulsions entirely ceased, but the same fits of nervous excitement still continued, which were followed by deep sleep and stertorous breathing, instead of the convulsions as heretofore.

The pulse throughout the day varied from 80 to 100 per minute. Respiration 10 to 12, skin warm, with free perspiration.

April 26th.—Pulse 120, rather feeble; respiration 8, and very labored. the nervous excitement followed by sleep, the same as before. Nurses were required to prevent the patient from leaving her bed during the fits of excitement.

The treatment consisted of chicken broth and brandy; cold water was also allowed, as the patient manifested great thirst.

April 27th.—Patient evidently sinking; no other change perceptible. Gave carb. ammonia in addition to yesterday's treatment.

April 28th.—Entire absence of nervous excitement; patient remaining quiet; respiration 24, pulse 130, very feeble. Continue the stimulating and supporting treatment.

Died at 9 P. M. No autopsy being made, therefore I submit the following remarks:

1st.—The exciting cause was evidently an increased pressure on the pelvic nerves, convulsions being produced through reflex action.

2d.—The convulsions, after the birth of the child, was evidently the continuation of shock on the nervous centres, and might have been prevented, by a continuation of the anæsthesia, till the system had somewhat regained its wonted vigor, which was fully proven by the entire subsequent arrest, by treatment.

3d.—The slow, labored and stertorous respiration during sleep might lead us to suspect cerebral compression.

Lastly.—The new train of symptoms, which took place on the last day to-wit: Increased respiration, feeble and rapid pulse, great prostration and death by asthenia, together with the blood-letting, and the length of time the patient was under the influence of chloroform, might all be considered as favorable symptoms and circumstances favoring the formation of heart-clot; all of which amounts only to mere conjecture, in the absence of direct evidence.

The treatment was begun on the principle, which many authors advocate, the removing of a hyperemic condition of the system, without having the least effect in arresting or mitigating the severity or number of convulsions.

Antispasmodics and anodynes were used, conjointly with blood-letting, without benefit; but after the birth of the child these remedies were indispensably useful in controlling the nervous excitement, to which I have alluded in my notes.

In conclusion I submit the notes of the above case to the consideration of the profession, believing the treatment by the inhalation of chloroform to be the most rational and efficient. Also believing that, had this been the treatment adopted at the beginning, and strictly adhered to throughout labor, nearly eight convulsions might have been avoided, and possibly with a different result.

